# Modelling the Potential Risk of Infection Associated with *Listeria monocytogenes* in Irrigation Water and Agricultural Soil in Two District Municipalities in South Africa

**DOI:** 10.3390/microorganisms10010181

**Published:** 2022-01-14

**Authors:** Chidozie Declan Iwu, Chinwe Juliana Iwu-Jaja, Rami Elhadi, Lucy Semerjian, Anthony Ifeanyin Okoh

**Affiliations:** 1 SAMRC Microbial Water Quality Monitoring Centre, University of Fort Hare, Alice 5700, South Africa; aokoh@sharjah.ac.ae; 2 Applied and Environmental Microbiology Research Group, Department of Biochemistry and Microbiology, University of Fort Hare, Alice 5700, South Africa; 3 Division of Health Systems and Public Health, Department of Global Health, Stellenbosch University, Stellenbosch 7600, South Africa; chinwelolo@gmail.com; 4 Department of Environmental Health Sciences, University of Sharjah, Sharjah P.O. Box 27272, United Arab Emirates; relhadi@sharjah.ac.ae (R.E.); lsemerjian@sharjah.ac.ae (L.S.)

**Keywords:** *Listeria monocytogenes*, listeriosis, irrigation water, agricultural soil, public health, QMRA

## Abstract

*Listeria monocytogenes* (*L. monocytogenes*) is the etiologic agent of listeriosis which significantly affects immunocompromised individuals. The potential risk of infection attributed to *L. monocytogenes* in irrigation water and agricultural soil, which are key transmission pathways of microbial hazards to the human population, was evaluated using the quantitative microbial risk assessment modelling. A Monte Carlo simulation with 10,000 iterations was used to characterize the risks. High counts of *L. monocytogenes* in irrigation water (mean: 11.96 × 10^2^ CFU/100 mL; range: 0.00 to 56.67 × 10^2^ CFU/100 mL) and agricultural soil samples (mean: 19.64 × 10^2^ CFU/g; range: 1.33 × 10^2^ to 62.33 × 10^2^ CFU/g) were documented. Consequently, a high annual infection risk of 5.50 × 10^−2^ (0.00 to 48.30 × 10^−2^), 54.50 × 10^−2^ (9.10 × 10^−3^ to 1.00) and 70.50 × 10^−2^ (3.60 × 10^−2^ to 1.00) was observed for adults exposed to contaminated irrigation water, adults exposed to contaminated agricultural soil and children exposed to agricultural soil, respectively. This study, therefore, documents a huge public health threat attributed to the high probability of infection in humans exposed to *L. monocytogenes* in irrigation water and agricultural soil in Amathole and Chris Hani District Municipalities in the Eastern Cape province of South Africa.

## 1. Introduction

*L. monocytogenes* is a ubiquitous Gram-positive bacterium naturally occurring in agrarian environments including soil, manure and water [1]. It is psychrotrophic, having the ability to grow below 7 °C under both aerobic and anaerobic conditions and at low levels of water activity in a wide range of pH (4.0–6.0) [2]. It can also persist under high salt concentration, hydrostatic pressure, oxidative stress and extreme energy levels [3]. These tenacious characteristics make *L. monocytogenes* a potential hazard in the food sector and a significant public health burden [4]. Once exposed, this pathogen causes severe illness due to its ability to induce its own phagocytosis and intracellular replication, cross the epithelial barriers and invade other healthy cells using virulence factors like hemolysins, phospholipases, internalins and surface protein actin A [5,6]. As a result, *L. monocytogenes* has become a model to study intracellular pathogens [7].

*L. monocytogenes* represents an unprecedented microbial hazard with public health significance, despite not being recognized as a popular cause of foodborne related illnesses [8]. It causes the disease listeriosis, which may be mild in healthy individuals or invasive in immunocompromised individuals, pregnant women, children and the elderly. While the mild form of listeriosis is characterized by flu-like symptoms, vomiting and diarrhoea, the invasive form is characterized by septicemia, meningitis, fetal infection, abortion and death [9]. The fatality rate of listeriosis is usually high, ranging from 20% to 40%, especially among the high-risk groups [10]. In South Africa, an astounding total of 1024 cases of listeriosis with a 28.6% fatality rate was recorded between 1 January 2017 and 24 April 2018, making it the largest listeriosis outbreak reported by the World Health Organization (WHO) [11].

The incidence rate of listeriosis in the population is generally low compared to other foodborne diseases. However, the distribution of *L. monocytogenes* in the environment is wide, with a high recovery rate in foods [8]. *L. monocytogenes* has been isolated from several ready-to-eat (RTE) foods such as ice cream [12], cheese [13], fish [14], meat pâté and meat products [15]. It has also been isolated from minimally processed fruits and vegetables such as caramel-apples [16], cantaloupe, bean sprouts [17], frozen vegetables [18] and packaged salads [19]. This is attributed to its ubiquitous nature in the food processing and agricultural environments [20].

*L. monocytogenes* is widely dispersed in the agroecosystem, especially in irrigation water sources, agricultural soil, vegetation and organic matters [21,22,23]. Many irrigation water sources harbour numerous enteric pathogens like *L. monocytogenes* which are introduced by pollutants like faecal materials, sewage, and soil particles [24]. The soil is also a known environmental niche for *L. monocytogenes*. This pathogen has been recovered from various soil samples in different locations, including mountainous areas, forest areas and grazing lands [1]. In the soil, *L. monocytogenes* exist naturally as saprophytes, but become pathogenic once present in human and animal cells [25].

The presence of *L. monocytogenes* in irrigation water and agricultural soil is detrimental to food safety and public health. A systematic risk assessment is therefore required to quantitatively predict the health risks posed by the presence of this pathogen in irrigation water and agricultural soil using information like the nature of the pathogen, its exposure routes and the health effects associated with the exposure [26]. To that effect, a quantitative microbial risk assessment (QMRA) modelling was carried out to predict the risks of infection attributed to *L. monocytogenes* in irrigation water and agricultural soil samples collected from Amathole and Chris Hani District Municipalities, Eastern Cape Province, South Africa. To the best of our knowledge, this is the first QMRA modelling of *L. monocytogenes* in irrigation water and agricultural soil to be carried out in the Province.

## 2. Materials and Methods

### 2.1. Study Area

**Figure 1:**
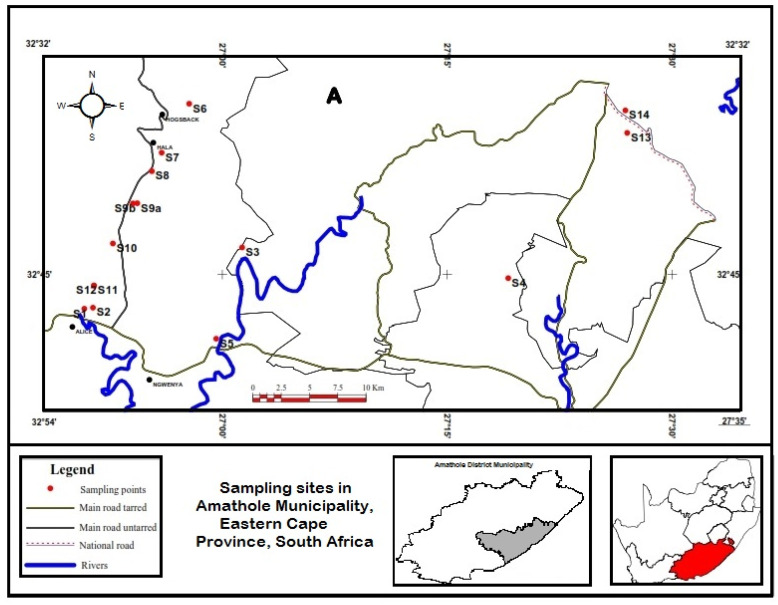
Map showing the sampling sites in Amathole District Municipality (grey area), Eastern Cape Province (red area), South Africa.

**Figure 2:**
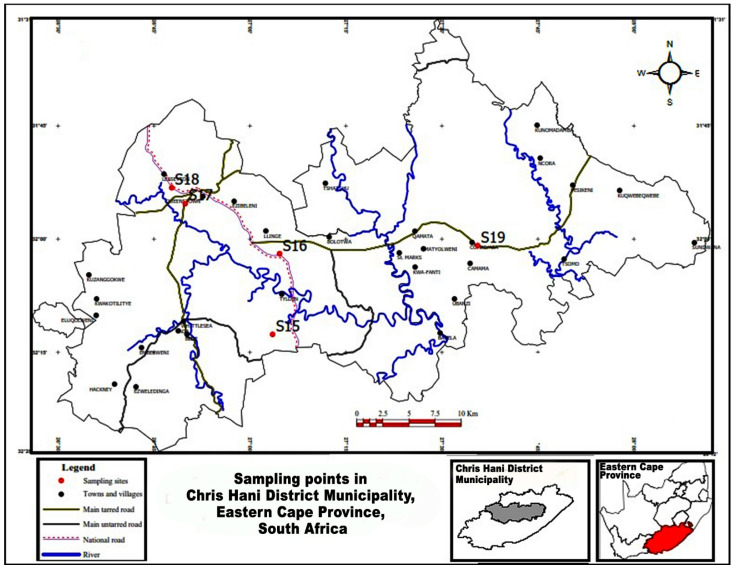
Map showing the sampling sites in Chris Hani District Municipality (grey area), Eastern Cape Province (red area), South Africa.

This study was conducted in two District Municipalities of the Eastern Cape Province, South Africa. The Province is the second largest province in the country and agriculture is one of its major industries. Samples were collected from 14 sampling sites in Amathole District Municipality, which is situated in the central part of the province, as shown in Figure 1. Samples were also collected from five sampling sites in the Chris Hani District Municipality situated in the northern region of the province, as shown in Figure 2. As of 2016, Amathole District Municipality was made up of 862,000 people (12.3% of Eastern Cape population and 1.55% of South African population) [27] while Chris Hani District Municipality was made up of 849,000 people (12.0% of Eastern Cape population and 1.5% of South Africa population) as of 2017 [28].

### 2.2. Microbiological Analysis

Using the grab sampling technique, a total of 19 irrigation water samples and 13 agricultural soil samples were aseptically collected from the sampling sites in sterile sample bottles and plastic bags, respectively. The sampling was done in triplicate across all of the sampling sites. The collected samples were transported on ice to the laboratory for microbiological analysis.

To determine the viable counts of *L. monocytogenes*, all the samples were subjected to serial dilution, which was then followed by membrane filtration for irrigation water samples and the spread plate culture method for soil samples as described in our previous report [29]. All the analyses were done in triplicates and the culture was done using Chromogenic Listeria Agar (ISO) Base (Oxoid Ltd., Hampshire, UK) supplemented with OCLA (ISO) differential supplement (Oxoid Ltd., UK) and the OCLA (ISO) selective supplement (Oxoid Ltd., UK). The concentration of *L. monocytogenes* was expressed in CFU/100 mL of irrigation water samples and CFU/g of agricultural soil samples.

The polymerase chain reaction (PCR) was used to confirm the identities of the isolates by targeting the *prs* gene (specific for genus) and *prf*A gene (specific for species) following the description of [30]. Nine virulence genes commonly associated with *L. monocytogenes* were screened for in the confirmed isolates using PCR assays as described by [31]. These include *inlA*, *inlB*, *inlC*, *inlJ*, *actA*, *hlyA*, *plcA*, *plc*B, and *iap. L. monocytogenes* ATCC 9525 (ATCC; Manassas, VA, USA) was included in the PCR as a control strain. The primer sequences, the expected amplicon sizes and the cycling conditions used in all the PCR experiments are shown in Table 1.

### 2.3. Microbial Risk Modelling

The probability of infection associated with *L. monocytogenes* in irrigation water and agricultural soil from Amathole and Chris Hani DMs was estimated using a four-step science-based approach including hazard identification, hazard characterization, exposure assessment and risk characterization as described by Codex Alimentarius Commission (CAC) [36].

#### 2.3.1. Hazard Identification

*L. monocytogenes* was selected to predict the health risks associated with contaminated irrigation water and agricultural soil. This pathogen was selected due to its high prevalence in the agricultural and food processing environments and its ability to be transmitted to the food chain where it can instigate severe foodborne related listeriosis. *L. monocytogenes* was also selected due to its significance in South Africa, having been implicated in the most severe form of listeriosis outbreak ever experienced globally [37].

#### 2.3.2. Hazard Characterization

Hazard characterization was carried out to access the negative health outcomes associated with the occurrence of *L. monocytogenes* in irrigation water and agricultural soil, based on the assumption that a single cell of *L. monocytogenes* would cause an infection. This analysis defines the relationship between the dose of *L. monocytogenes* and the corresponding negative health effects on the exposed population [38]. The following equation [39] was used to determine the ingestion dose of *L. monocytogenes*.

D = (I_v_ × M_c_) (1)

where D represents the ingestion dose of *L. monocytogenes*, I_v_ represents the ingested volume of irrigation water and agricultural soil and M_c_ represents the mean viable counts of *L. monocytogenes*.

An “exponential dose-response model” [40] was used to evaluate the risk linked to *L. monocytogenes* as shown in the following equation;

P_inf_ = 1 − exp ^−rD^ (2)

where P_inf_ represents the probability of infection that will occur in an individual exposed to a particular dose (D) of *L. monocytogenes*. D represents the ingestion dose of *L. monocytogenes* and r represents the probability that a single cell of *L. monocytogenes* will cause invasive listeriosis. In this equation, r is 1.91 × 10^−10^ which is constant for *L. monocytogenes* [41].

#### 2.3.3. Exposure Assessment

Exposure assessment was carried out to describe the possible ways by which susceptible human populations are exposed to *L. monocytogenes* in irrigation water and agricultural soil as well as to model the number of exposures that exist between humans and *L. monocytogenes*. Factors such as the counts of pathogen in the environmental matrix, ingested volumes of the matrix, the viability of the pathogen, and the recovery efficacy of the methods were considered in the Exposure (E) assessment using the following equation [42]:

E = CR^−1^ IM (3)

where E represents Exposure, C represents the counts of *L. monocytogenes* per 100 mL of irrigation water samples or per gram of soil samples, R represents the recovery efficacy of the isolation method, I represents the fraction of *L. monocytogenes* capable of causing severe infection, and M represents the amount of irrigation water and soil ingested unintentionally per day. Recovery efficacy (R) was considered to prevent the underestimation of the concentration of the pathogen as well as the exposure [42]. It was estimated using the equation below:

R = (Po − P/Po) × 100(4)

where “Po” represents the presumptive counts of *L. monocytogenes* isolates in irrigation water and agricultural soil samples and “P” represents the confirmed isolates following cultural and molecular methods. The parameters inputted for exposure assessment are shown in Table 2

#### 2.3.4. Risk Characterization

Risk characterization was done to predict the annuitized risk of infection based on hazard identification, hazard characterization and exposure assessment using the annuitized probability of infection (P_inf/y_) equation [39] as shown below.

P_inf/y_ = 1 − (1 − P_inf_)^E^ (5)

where P_inf/y_ represents the yearly probability of infection, P_inf_ represents the probability of infection due to a single exposure to an ingested dose (D) of *L. monocytogenes* and E represents the exposure.

The risk associated with a single exposure to *L. monocytogenes* was evaluated using a Monte Carlo simulation with 10,000 iterations. The modelling was performed using R software version 3.0.3 (Development Core Team from Vienna, Austria) with the application of the R package (fitdistrplus) to fit the distribution of pathogen concentrations.

## 3. Results and Discussion

### 3.1. Hazard Identification and Concentration of L. monocytogenes in the Samples

**Figure 3:**
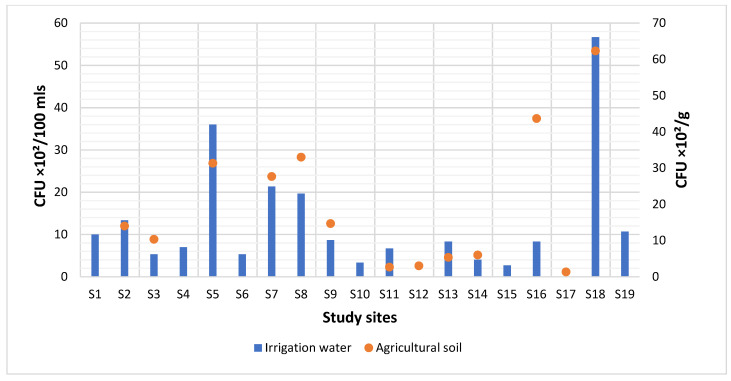
The microbial counts of *L. monocytogenes* in irrigation water and agricultural soil samples. Agricultural soil samples were not collected from S1, S4, S6, S10, S15 and S19.

In this study, the mean concentration of *L. monocytogenes* in irrigation water samples was 11.96 × 10^2^ CFU/100 mL ranging from 0 to 56.67 × 10^2^ CFU/100 mL as shown in Figure 3. This exceeded 0.0 CFU/100 mL standard set by the South African Department of Water Affairs (DWAF) for faecal coliforms in domestic water [45] and ≤100 CFU/100 mL standard set by the World Health Organization (WHO) for coliforms in wastewater used for agriculture and aquaculture [46]. This suggests that the irrigation waters within the study sites are not of great quality for agricultural activities, hence posing health risks to the exposed population. The findings are also consistent with our previous study which assessed the prevalence of *Listeria* spp. in river and irrigation water in the Eastern Cape Province of South Africa [47]. A higher mean concentration of *L. monocytogenes* was recorded in the agricultural soil samples, estimated at 19.64 × 10^2^ CFU/g and ranging from 1.33 × 10^2^ CFU/g to 62.33 × 10^2^ CFU/g, as shown in Figure 3. This is not surprising, as *L. monocytogenes* is widely dispersed in the soil, also posing human health risks.

Of the 117 presumptive *L. monocytogenes* recovered from irrigation water samples and 183 presumptive *L. monocytogenes* isolated from agricultural soil samples, eight (6.8%) and 12 (6.6%) isolates were confirmed, respectively, following the molecular analyses. These findings are lower than the results of a previous study that recovered 11.2% *L. monocytogenes* from surface water used to irrigate fresh produce, [48] and a study that recovered 4 to 11% *L. monocytogenes* from the soil of fresh leafy produce production fields [49]. Although *L. monocytogenes* is almost always recovered in low numbers, they can cause a high rate of infection, especially among the immunocompromised population.

Interestingly, each of the confirmed isolates in this study harbored all the screened virulence genes, indicating that they are highly pathogenic and can cause severe infections in potential exposed populations. This corroborates previous studies that assessed the prevalence of virulence genes in *L. monocytogenes* isolated from various food and environmental matrices [31,50,51,52,53]. Generally, virulence genes in *L. monocytogenes* are usually implicated in the various phases of infection induced by the pathogen. For instance, *hly*A, *prf*A and *act*A genes are involved with the spread of the pathogen between the cells of the host, the *inl*A, *inl*B, and *inl*J genes are involved with invasion and adhesion, the *plc*A and *plc*B genes facilitate the release of the pathogen from bound vacuoles and the *hly*A gene is also involved the release of the bacterial cells into the cells of the host [54].

### 3.2. Dose Modelling and Hazard Characterization

Table 3 shows the results of the dose modelling and hazard characterization of *L. monocytogenes* in irrigation water and agricultural soil in the study areas. A 2.30 × 10^−6^ daily risk (probability) of infection was estimated for adults ingesting 11.97 × 10^3^ doses of *L. monocytogenes* from irrigation water. The daily risk of infection was 1.10 × 10^−5^ at maximum ingestion dose of 56.67 × 10^3^ and 0.00 at minimum ingestion dose of 0.00. These estimates were based on the assumption that adults intentionally or unintentionally ingest 10 mL of contaminated irrigation water per day [43]. A higher daily risk of infection (4.12 × 10^−3^) attributed to *E. coli* in unprotected spring water was recorded in a previous study assuming the ingestion volume was 500 mL [55]. This shows that ingestion volume is correlated to the daily risk of infection. The probability of infection in children was not recorded in this study because the parameter for the ingested volume of irrigation water by children was not available.

Also, a 1.90 × 10^−5^ daily risk of infection was recorded for adults ingesting 98.21 × 10^3^ doses of *L. monocytogenes* from agricultural soil. The probability of infection was 1.30 × 10^−6^ at minimum ingestion dose of 6.67 × 10^3^ and 6.00 × 10^−5^ at maximum ingestion dose of 311.67 × 10^3^. These estimates were based on the assumption that adults intentionally or unintentionally ingest 50 mg of contaminated soil per day [44]. These estimates were lower than those obtained in a previous study for other enteric pathogens like *E. coli* (6.38 × 10^−2^) and *Salmonella* spp. (2.43 × 10^−1^) in contaminated soil [55]. In children, a 3.80 × 10^−5^ probability of infection was recorded at an ingestion dose of 196.41 × 10^3^. The probability of infection was 2.50 × 10^−6^ at minimum ingestion dose of 13.33 × 10^3^ and 1.20 × 10^−4^ at maximum ingestion dose of 623.33 × 10^3^. These estimates were also based on the assumption that children intentionally or unintentionally ingest 100 mg of contaminated soil per day [44].

It has been shown that the probability of infection attributed to pathogens in the environment depends on certain factors such as the pathogenicity of the pathogen, the ingestion dose of the pathogen, and the exposure routes to the pathogen [55].

### 3.3. Exposure Assessment

**Figure 4:**
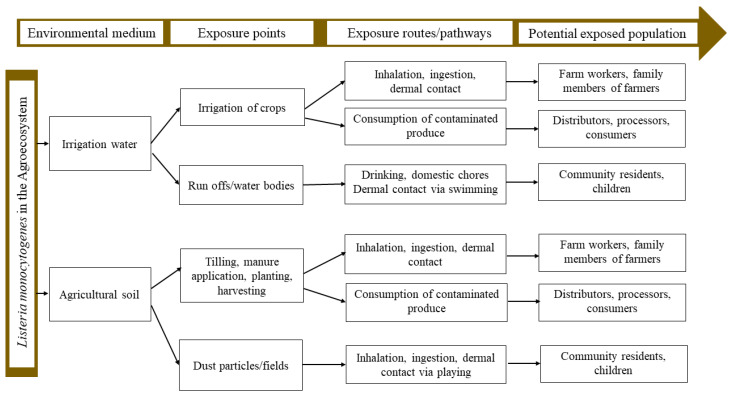
The human exposure patterns showing possible ways humans can be exposed to *L. monocytogenes* in irrigation water and agricultural soil.

In this study, the patterns of human exposure to *L. monocytogenes* in irrigation water and agricultural soil is shown in Figure 4. One of the common routes of exposure is via the ingestion of fresh produce contaminated by *L. monocytogenes* in irrigation water and agricultural soil. This potentially puts the lives of farmers, their family members, consumers, distributors and processors in danger. Steele et al. indicated that contaminated irrigation water is a significant source of fresh produce contamination, correlating to the rise in the frequency of foodborne infections [56]. Smith et al. also indicated that *L. monocytogenes* in the soil can be transferred to fresh produce via splashes of soil during rainfall or irrigation, human activities, direct contact of plant surfaces with the soil and through machinery [57]. Other possible exposure routes include the unintentional ingestion, inhalation and dermal contact of contaminated irrigation water and soil particles, therefore putting the lives of farmers, their family members and co-workers at risk. Furthermore, children and community members playing in the soil, swimming and collecting water from irrigation water sources for other domestic purposes have their lives at risk when exposed to *L. monocytogenes* via ingestion, dermal contact and inhalation.

Considering the parameters inputted for the evaluation of exposure, a 12.87 × 10^3^ exposure with a range of 0.00 to 60.93 × 10^3^ was documented for adults exposed to *L. monocytogenes* in irrigation water as shown in Table 4. Alternatively, a 105.60 × 10^3^ exposure with a range of 7.17 × 10^3^ to 335.13 × 10^3^ was documented for adults exposed to *L. monocytogenes* in agricultural soil, while a 211.19 × 10^3^ exposure with a range of 14.34 × 10^3^ to 670.25 × 10^3^ was recorded for children exposed to *L. monocytogenes* in agricultural soil as shown in Table 4. Numerically, this indicates that people, especially children are more likely to be exposed to *L. monocytogenes* in agricultural soil than in irrigation water, thus increasing their risks of infection.

### 3.4. Risk Characterization

The annual risk of infection in adults exposed to *L. monocytogenes* from irrigation water was 5.50 × 10^−2^ with a range of 0.00 to 48.30 × 10^−2^ as shown in Table 5. This exceeded the WHO permissible standard for the annual tolerable reference level of human health risk attributed to drinking water (1 × 10^−4^) [58] and that attributed to excreta and greywater used for agricultural activities (1 × 10^−6^ DALY) [59]. This suggests that the irrigation water in this study is of unacceptable quality and poses health risks to the exposed population. A similar finding was recorded in a previous study that used rotavirus as a model organism to estimate the annual risk of infection attributed to irrigation water [60]. Furthermore, a QMRA simulation predicted a high mean risk of infection (8.10 × 10^−6^ per month) attributed to *L. monocytogenes* in RTE vegetables [61]. The finding was attributed to the pervasiveness of *L. monocytogenes* in the environment, which is consistent with our findings.

The annual risk of infection in adults exposed to *L. monocytogenes* from agricultural soil was 54.50 × 10^−2^ with a range of 9.10 × 10^−3^ to 1.00, while the annual risk of infection in children exposed to *L. monocytogenes* from agricultural soil was 70.50 × 10^−2^ with a range of 3.60 × 10^−2^ to 1.00 as shown in Table 5. A similar annual risk of 5.47 × 10^−1^ attributed to other enteric pathogens like *E. coli* in open space contaminated soil, which is a playground for children, was documented in a previous study [55]. However, a much higher annual risk of 9.65 × 10^−1^ attributed to *Salmonella* spp. in the same soil was documented [55].

The odds of listeriosis occurring while infected with *L. monocytogenes* is low. However, this pathogen is more likely to cause more devastating effects on pregnant women and their neonates, elderly ones and those with a weakened immune system [62]. Unfortunately, the Eastern Cape Province of South Africa is the second-largest province, yet the most impoverished [63]. The province has a high burden of diseases such as HIV, tuberculosis (TB), HIV/TB coinfection and multidrug-resistant TB (MDR-TB) [64]. This predisposes the residents to the worst outcomes of listeriosis, whose probability of occurring is high and thus calls for urgent attention from relevant stakeholders and risk managers.

To the best of our knowledge, this study was the first to be conducted in the Eastern Cape Province, South Africa. It was not, however, without limitations. The study employed a more general approach by assuming that every exposed individual will ingest the same amount of contaminated irrigation water and agricultural soil, thus having the same risk of infection. This is, however, not always the case, since certain factors such as age and behaviour can affect the amount of contaminated irrigation water and agricultural soil that is ingested. Moreover, certain factors such as age, immune status, infectious dose, gender and co-morbidities can influence the outcome of exposure to pathogens in the environmental matrix [58]. Since the model predicts the risk per annum, the sample size was low, and the grab sampling did not factor in potential exposure fluctuations due to seasonality.

## 4. Conclusions

The findings of this study indicated that the concentration of *L. monocytogenes* in irrigation water and agricultural soil samples collected from Amathole and Chris Hani District Municipalities were high, consequently leading to a high annual risk of infection among the exposed population. This poses a huge public health risk and requires urgent control measures. Outcomes from the study may help risk managers apply appropriate and timely interventions to control the health risks.

## Figures and Tables

**Table 1:** Primer sequence, expected amplicon sizes and cycling conditions used in the detection of *L. monocytogenes* and screening of virulence genes.

Primer Sequence (5′-3′)	Target Genes	Cycling Conditions	Amplicon Size (bp)	References
F: GCTGAAGAGATTGCGAAAGAAG R: CAAAGAAACCTTGGATTTGCGG	*prs*	5 min 95 °C 35 [30 s 94 °C, 90 s 60 °C, 90 s 72 °C] 5 min 2 °C	370	[30]
F: GATACAGAAACATCGGTTGGC R: GTGTAATCTTGATGCCATCAG	*prf*A	5 min 95 °C 35 [30 s 94 °C, 90 s 60 °C, 90 s 72 °C] 5 min 2 °C	274	[30]
inlAF: CCTAGCAGGTCTAACCGCAC inlAR: TCGCTAATTTGGTTATGCCC	*inl*A	5 min 94 °C 35 [35 s, 94 °C; 30 s, 52 °C; 1 min, 72 °C] 10 min 72 °C	256	[32]
inlBF: TGATGTTGATGGAACGGTAAT inlBR: CTCGTGGAAGTTTGTAGATGC	*inl*B	5 min 94 °C 35 [35 s, 94 °C; 30 s, 52 °C; 1 min, 72 °C] 10 min 72 °C	272	[31]
inlCF: AATTCCCACAGGACACAACC inlCR: CGGGAATGCAATTTTTCACTA	*inl*C	5 min 94 °C 35 [35 s, 94 °C; 30 s, 52 °C; 1 min, 72 °C] 10 min 72 °C	517	[33]
inlJF: TGTAACCCCGCTTACACAGTT inlJR: AGCGGCTTGGCAGTCTAATA	*inl*J	5 min 94 °C 35 [35 s, 94 °C; 30 s, 52 °C; 1 min, 72 °C] 10 min 72 °C	238	[33]
actAF: CCAAGCGAGGTAAATACGGGA actAR: GTCCGAAGCATTTACCTCTTC	*act*A	5 min 94 °C 35 [35 s, 94 °C; 30 s, 52 °C; 1 min, 72 °C] 10 min 72 °C	650	[34]
hlyF: ATCATCGACGGCAACCTCGGAGAC hlyR: CACCATTCCCAAGCTAAACCAGTGC	*hly*A	5 min 94 °C 35 [35 s, 94 °C; 30 s, 52 °C; 1 min, 72 °C] 10 min 72 °C	404	[31]
plcAF: CTCGGACCATTGTAGTCATCTT plcAR: CACTTTCAGGCGTATTAGAAACGA	*plc*A	5 min 94 °C 35 [35 s, 94 °C; 30 s, 52 °C; 1 min, 72 °C] 10 min 72 °C	326	[34]
plcBF: AATATTTCAATCAATCGGTGGCTGA plcBR: GGGTAGTCCGCTTTCGCTCTT	*plc*B	5 min 94 °C 35 [35 s, 94 °C; 30 s, 52 °C; 1 min, 72 °C] 10 min 72 °C	289	[31]
iapF: ACAAGCTGCACCTGTTGCAG iapR: TGACAGCGTGTGTAGTAGCA	*iap*	5 min 94 °C 35 [35 s, 94 °C; 30 s, 52 °C; 1 min, 72 °C] 10 min 72 °C	131	[35]

**Table 2:** Parameters inputted for exposure assessment in adults and children.

Irrigation Water			Agricultural Soil		
Parameter	Data	Source	Parameter	Data	Source
Concentration (C) of *L. monocytogenes* (CFU/100 mL)	Min: 0.00 Mean: 11.96 × 10^2^ Max: 56.67 × 10^2^	This study	Concentration (C) of *L. monocytogenes* (CFU/g)	Min: 1.33 × 10^2^ Mean: 19.64 × 10^2^ Max: 62.33 × 10^2^	This study
Recovery efficiency (R) (%)	93	This study	Recovery efficiency (R) (%)	93	This study
Proportion (I) of *L. monocytogenes* capable of causing severe infection (%)	100	This study	Proportion (I) of *L. monocytogenes* capable of causing severe infection (%)	100	This study
Amount (M) of water ingested during farming (mL/day)	10	[43]	Amount (M) of soil and dust ingested by adults (mg/day)	50	[44]
Amount (M) of water ingested by children during farming	Not given		Amount (M) of soil and dust ingested by children (mg/day)	100	[44]

Min: Minimum, Max: Maximum.

**Table 3:** The daily probability of infection based on hazard characterization in irrigation water and agricultural soil samples.

Parameter	Irrigation Water			Agricultural Soil		
	Min	Mean	Max	Min	Mean	Max
Ingestion dose (D) in adults	0.00	11.97 × 10^3^	56.67 × 10^3^	6.67 × 10^3^	98.21 × 10^3^	311.67 × 10^3^
Ingestion dose (D) in children	-	-	-	13.33 × 10^3^	196.41 × 10^3^	623.33 × 10^3^
Probability of infection (P_inf_) in adults (daily risk)	0.00	2.30 × 10^−6^	1.10 × 10^−5^	1.30 × 10^−6^	1.90 × 10^−5^	6.00 × 10^−5^
Probability of infection (P_inf_) in children (daily risk)	-	-	-	2.50 × 10^−6^	3.80 × 10^−5^	1.20 × 10^−4^

Min: Minimum, Max: Maximum.

**Table 4:** The exposure parameters in adults and children.

	Irrigation Water	Agricultural Soil
Parameter	Data	Data
Exposure (E) in adults	Min: 0.00 Mean: 12.87 × 10^3^ Max: 60.93 × 10^3^	Min: 7.17 × 10^3^ Mean: 105.60 × 10^3^ Max: 335.125 × 10^3^
Exposure (E) in children	Not determined	Min: 14.34 × 10^3^ Mean: 211.19 × 10^3^ Max: 670.25 × 10^3^

Min: Minimum, Max: Maximum.

**Table 5:** The annual risk of infection due to ingestion of *Listeria monocytogenes* in irrigation water and agricultural soil.

Parameter	Irrigation Water			Agricultural Soil		
	Min	Mean	Max	Min	Mean	Max
Annual risk (P_inf/y_) in adults	0.00	5.50 × 10^−2^	48.30 × 10^−2^	9.10 × 10^−3^	54.50 × 10^−2^	1
Annual risk (P_inf/y_) in children	-	-	-	3.60 × 10^−2^	70.50 × 10^−2^	1

Min: Minimum, Max: Maximum.
